# Chicago Academy of the Medical Sciences

**Published:** 1859-04

**Authors:** 


### MISCELLANEOUS MEDICAL INTELLIGENCE.

### CHICAGO ACADEMY OF THE MEDICAL SCIENCES.

A new medical society has recently been organized in our city, with the above title. Judging from the harmony and zeal manifested at its former meetings, and also from the high character of the gentlemen composing it, we have not the least hesitation in saying, that it will be an entire success, fulfilling the expectations of its most sanguine friends. The profession of Chicago have long felt the need of such an institution as the present organization promises to be, and it is to be hoped that the medical fraternity will unite in placing it on a basis equal to that of the N. Y. Academy of Medical Science, and other kindred societies in eastern cities. They propose to secure a charter at the next sitting of the Legislature, thus securing for themselves a permanent foundation. One of the main features of the organization is the formation of a library and cabinet.

The Academy meets on the first Monday of every month, at the lecture room of Sloan's Commercial College.

The following is a list of the officers:

| | |
|---|---|
| *President,* | De Laskie Miller, M.D. |
| 1*st Vice-President,* | J. N. Graham, M.D. |
| 2*d* " " | J. R. Gore, M.D. |
| 3*d* " " | E. C. Rogers, M.D. |
| *Recording Secretary,* | S. C. Blake, M.D. |
| *Corresponding Secretary,* | E. L. Holmes, M.D. |
| *Treasurer,* | R. C. Hamil, M.D. |

*Trustees.*

James Bloodgood, M.D. J. P. Ross, M.D.
Ephraim Ingalls, M.D.

*Committee on Admissions.*

Thomas Bevan, M.D., Wm. Scott Denniston, M.D.
S. Schneider, M.D.

*Committee on Medical Ethics.*

H. A. Johnson, M.D., James Bloodgood, M.D.
David Rutter, M.D.

*Committee on Medical Education.*

Charles Duck, M.D., Ephraim Ingalls, M.D.
E. L. Holmes, M.D.

*Delegates to American Medical Association.*

Dr. S. C. Blake, Dr. J. L. Page, and Dr. J. N. Graham.

*Delegates to Indiana State Medical Society.*

Dr. J. L. Page. Dr. J. N. Graham.

